# Governing Children

**Published:** 1854-07

**Authors:** 


### GOVERNING CHILDREN.

We have known religious parents who purposely checked, and crossed, and disappointed their children, as a system of home education, in order, as they alleged, to break the natural will, and thus make it easier for them in after life, to deny self and practice virtue. When we see such a course pursued, we think of the child's remark, when asked why a certain tree grew crooked—"Somebody trod upon it, I suppose, when it was a little fellow."

Childhood needs direction and culture more than repression. There is a volume of sound truth in these lines:—

"He who checks a child with terror,
Stops its play and stills its song,
Not alone commits an error,
But a great and moral wrong.

"Give it play and never fear it,
Active life is no defect;
Never, never break its spirit,
Curb it only to direct.

"Would you stop the flowing river,
Thinking it would cease to flow?
Onward it must flow forever;
Better teach it where to go."

*Home Gazette.*

